# Case Report: Efficacy and safety of recombinant growth hormone therapy in a girl with Loeys–Dietz syndrome

**DOI:** 10.3389/fcvm.2024.1377510

**Published:** 2025-01-03

**Authors:** Kamil Dyrka, Aleksander Jamsheer, Michal Bartecki, Waldemar Bobkowski, Malgorzata Pawelec-Wojtalik, Justyna Rajewska-Tabor, Andzelika Tomaszewska, Justyna Balcerzak, Zuzanna Aniol, Marek Niedziela, Monika Obara-Moszynska

**Affiliations:** ^1^ Department of Pediatric Endocrinology and Rheumatology, Institute of Pediatrics, Doctoral School, Poznan University of Medical Sciences, Poznan, Poland; ^2^ Department of Medical Genetics, Poznan University of Medical Sciences, Poznan, Poland; ^3^ Department of Pediatric Cardiology, Institute of Pediatrics, Poznan University of Medical Sciences, Poznan, Poland; ^4^ 1st Department of Cardiology, Cardiac Magnetic Resonance Unit, Poznan University of Medical Sciences, Poznan, Poland; ^5^ Student Scientific Society of Pediatric Endocrinology, Poznan University of Medical Sciences, Poznan, Poland; ^6^ Department of Pediatric Endocrinology and Rheumatology, Institute of Pediatrics, Poznan University of Medical Sciences, Poznan, Poland

**Keywords:** Loeys-Dietz syndrome, TGFβR2, recombinant growth hormone, cardiovascular system, short stature

## Abstract

**Background:**

Loeys–Dietz syndrome (LDS) is a clinically and genetically heterogeneous, autosomal dominant aortic aneurysm syndrome with widespread systemic involvement. We present the case of a 16.5-year-old girl with LDS type 2 (LDS2) caused by a heterozygous pathogenic variant, c.1582C>T (p.Arg528Cys), in the transforming growth factor-beta receptor type 2 (TGFBR2) gene who was treated with recombinant growth hormone (rGH) due to coexisting GH deficiency (GHD). This case report (observational study) presents the efficacy of rGH therapy and the safety aspects of this treatment, including aortal imaging follow-up (echocardiography, ECHO). To our knowledge, this is the first investigation of the effects of long-term rGH treatment on aortic dimensions in an LDS patient.

**Case summary:**

LDS was recognized in the patient in the 2nd year of life. After the 3rd year of life, growth deceleration was observed. At age 6, GHD was recognized [the maximum GH after stimulation 7.2 ng/ml; insulin-like growth factor-1 (IGF-1), 35 ng/ml; *N*: 84–447]. At age 6.5 years, rGH was initiated (height standard deviation score, htSDS −2.4), which continued for up to 14.25 years (htSDS-1.4). Her height at 16.5 years was 155 cm. The dose of rGH was 0.025–0.028 mg/kg/day. After the age of 16 months, widening of the aortic root was observed via echocardiography. At nearly 16 years, due to dilated aortic root (Z score +5.95), the girl underwent a plastic operation on the aorta, which had a satisfactory outcome. The patient's current status is stable, but the management of patients with LDS requires multidisciplinary cooperation due to the many coexisting comorbidities.

**Conclusions:**

Although aortic dilatation occurs in most LDS patients, the possible influence of GH therapy on aortic size must be considered. However, whether IGF-1, the main biochemical marker of GH activity, can be independently associated with increased aortic diameter has not been determined. In addition to its growth-promoting effect, the wide influence of GH on the human body, metabolic status, and muscle strength is also significant. The extremely low IGF-1 level before rGH therapy in the present patient and the strict monitoring of the IGF1/IGFBP3 ratio during rGH administration seem to be safe and beneficial for therapy.

## 1 Introduction

Loeys–Dietz syndrome (LDS) was first described in 2005 (1). Clinical research that led to the diagnosis of LDS focused on probands with three main perturbations: hypertelorism, cleft palate, and aortic or arterial aneurysms. LDS is a disease with an autosomal dominant inheritance, and approximately 70% of cases result from a *de novo* pathogenic variant (2). The disorder is genetically heterogeneous and caused by mutations in the genes of the transforming growth factor *β* (*TGF β*) signaling pathway: *TGFβR1* (LDS1), *TGFβR2* (LDS2), *SMAD3* (LDS3), *TGFβ2* (LDS4), *TGFβ3* (LDS5) and *SMAD2* (LDS6). TGFβ is a cytokine that plays an important role in many physiological processes. It participates in angiogenesis, apoptosis, and regulation of the amount of extracellular matrix protein. TGF-β pathway dysfunction leads to cardiovascular abnormalities and craniofacial and musculoskeletal manifestations. Mutations in *TGFβR2* are the most frequent cause of LDS (3–6).

**Figure 1:**
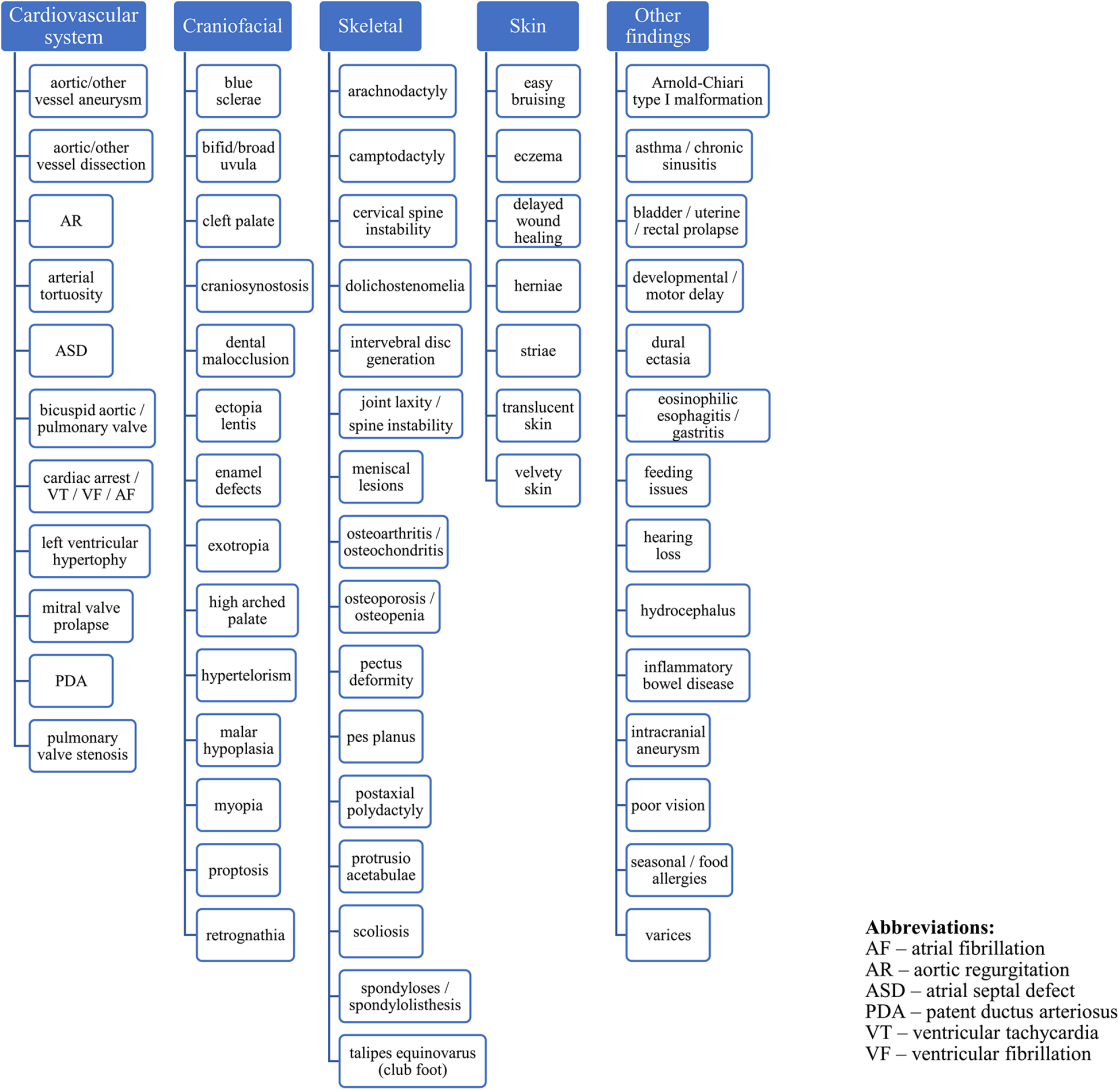
Clinical characterization of the LDS.

Clinical findings in four major systems characterize LDS: vascular, craniofacial, skeletal, and cutaneous. In the vascular system, the most frequent disorder is dilatation of the aortic root, which, if unnoticed, results in aortic dissection and rupture (7). The most common skeletal findings are pectus deformities, joint laxity and hypermobility. Craniofacial manifestations include micrognathia, retrognathia, shallow orbits, hypertelorism, and bifid uvula with or without cleft palate. Craniosynostosis and blue sclerae are also reported in LDS (1, 8). LDS is also associated with immunologic-related disorders: affected individuals exhibit food allergies, and LDS patients have a high incidence of asthma, rhinitis, and eczema (3). Gastroenteric manifestations include inflammatory bowel disease and nutritional problems (9, 10). The clinical features and complications of a subject with genetically confirmed LDS are summarized in Figure 1 (1, 11, 12). Due to multisystem disorders, some patients with LDS may present short stature and impaired development. Because this syndrome was first described nearly 15 years ago, the clinical experience is relatively poor, and possible therapeutic options still need to be discussed.

We present the case of a 16.5-year-old girl with LDS2 caused by the heterozygous pathogenic variant c.1582C>T (p.Arg528Cys) in the *TGFβR2* gene who was treated with recombinant growth hormone (rGH) for nearly eight years due to coexisting growth hormone deficiency (GHD). rGH therapy is used mainly in GHD patients. GH plays a key role in heart development and plays a positive role in maintaining the structure of the vascular endothelium. GH has a positive effect on aortic wall distensibility (13). Childhood GHD may restrict cardiac growth, and rGH treatment improves body size and cardiac mass, especially during the first year of therapy (14). However, whether IGF-1, the main biochemical marker of GH action (15), can be independently associated with increased aortic diameter has not been determined. Retrospective studies concerning rGH therapy in patients with Turner syndrome (TS), another genetic syndrome characterized by short stature and aortic dilation, do not suggest a cardiovascular risk (10, 16, 17).

This case series (observational study) reports the efficacy of rGH therapy and the safety of this treatment, including aortal imaging follow-up - echocardiography (ECHO). The safety of substitutional rGH therapy in LDS must be determined, especially given that cardiovascular complications are the most frequent anomalies in this syndrome. The benefits of rGH for LDS in addition to growth promotion, such as any positive effect on the skeleton, the stability of the joints, and the strength of the muscles, should be mentioned. To our knowledge, this is the first investigation of the effects of long-term rGH treatment on aortic dimensions in patients with LDS.

## 2 Materials and methods

### 2.1 Genetic analysis

Molecular genetic testing included polymerase chain reaction (PCR) amplification and bidirectional Sanger sequencing of all exons and flanking intronic regions of the *TGFβR1* and *TGFβR2* genes. The analytic methods used were previously described by Matyas et al. (18) and Jamsheer et al. (19).

### 2.2 Endocrinology

We calculated height standard deviation score (htSDS) for chronological age according with Polish references (20). We estimated bone age according to Greulich and Pyle (21).

### 2.3 Cardiology

ECHO examinations were performed during visits to the hospital cardiology clinic or outpatient clinic (throughout the patient's lifetime). Specialists in pediatric cardiology performed the surgeries.

ECHO was performed using a convex transducer. The aortic root and ascending aorta sizes were measured from the parasternal long-axis view. The sinus, sinotubular junction, and ascending aorta were measured in diastole using the leading-edge to leading-edge technique, and the obtained values were subsequently analyzed. Z scores were calculated using data developed by Gautier et al. (22). BSA was calculated using the DuBois formula.

**Figure 2:**
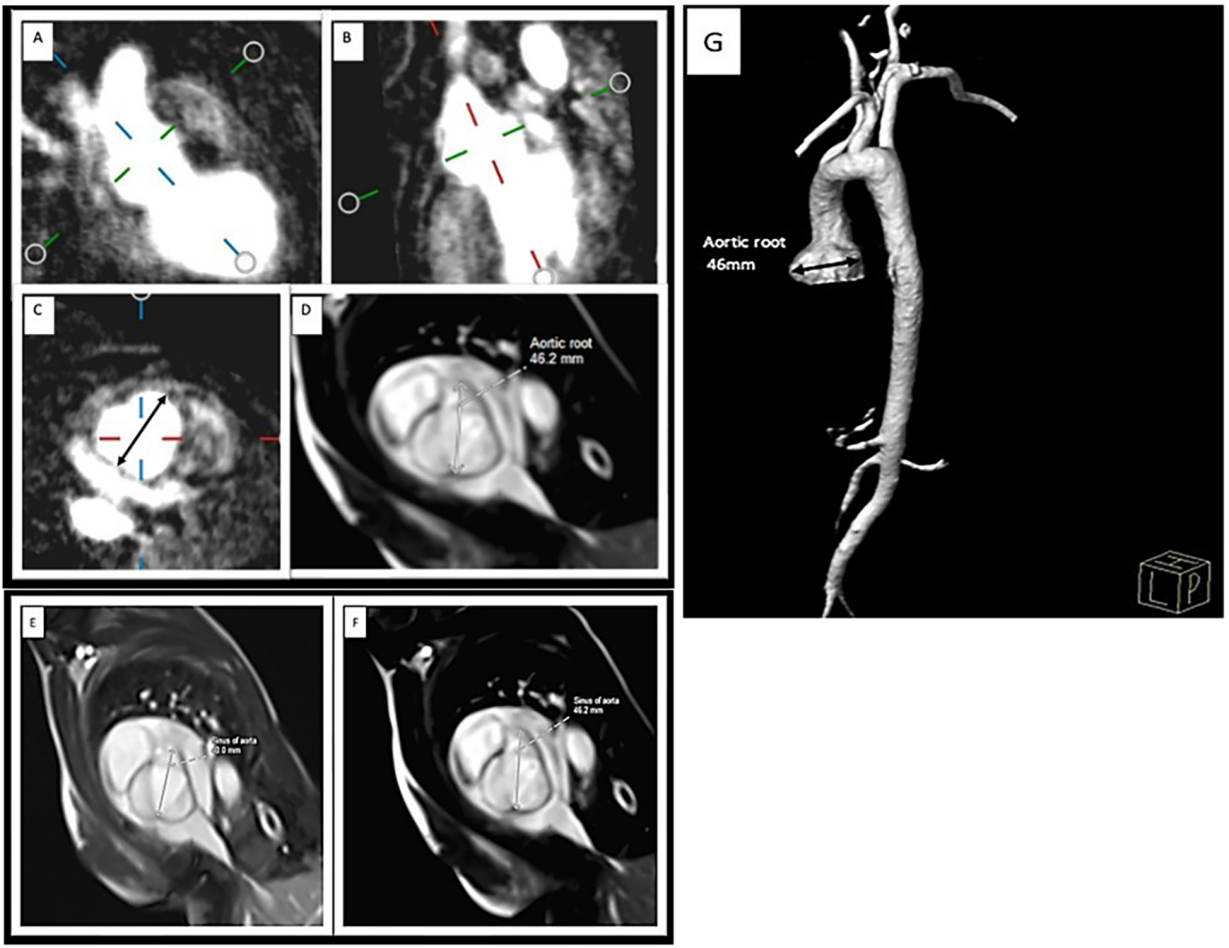
Measurement of the aortic root MRA: **(A,B)** perpendicular plane, **(C)** diameter of the ascending aorta in the transverse plane. CMR: **(D)** SSFP sequence of the aortic root. Aortic root diameter progression on CMR: **(E)** initial study (40 mm) and **(F)** examination after 11 months (46.2 mm). 3D reconstruction of the MRA image of the aorta in LDS: aortic sinus aneurysm. **(G)**.

Cardiac magnetic resonance (CMR) and 3D dynamic magnetic resonance angiography (MRA) were performed with a 1.5-tesla scanner (Siemens, Avanto) using a matrix coil for body and cardiac applications combined with a spinal coil. All the sequences were electrocardiogram (ECG)-gated during breath-holding on expiration. Angio-MR was performed using dynamic time-resolved angiography with interleaved stochastic trajectories (TWIST) after administering a contrast agent (0.1 mmol/kg, gadobutrol) followed immediately by a 20 ml saline flush. The duration of contrast agent injection was calculated after the administration of 1 ml of contrast agent. The sequence parameters were TR/TE 2.3/0.87 ms, field of view, 500 × 312.5 mm, slice thickness 1.5 mm, gap 0 mm, matrix size 384 × 223, and in-plane resolution 1.30 × 1.30 mm. The TWIST sequence was used for aortic measurements. The aortic diameter was measured at nine levels: the aortic root, sinotubular junction, ascending aorta, origin of the brachiocephalic artery, first transverse segment, second transverse segment, isthmic region, descending aorta, and thoracoabdominal aorta at the level of the diaphragm (Figure 2). All measurements were obtained with the dedicated software Medis Suite MR 3.0. The results for the aorta were compared against the ranges developed by Kaiser et al. (23). A standardized Z score for aortic diameter at each segment was calculated with an electronic calculator developed by Kaiser et al. (23), in which the diameter of each segment of the aorta and BSA were used.

## 3 Case report

The girl was born at 39 weeks of gestation by C-section and had a birth weight of 2,800 g, length of 51 cm, and an Apgar score of 10/10. Her parents (unrelated) and older brother (born three years before) did not present any health problems. Based on parental height, the target height was 154 cm.

Physical examination after delivery revealed cleft palate (surgery at 1.5 years old), craniosynostosis (premature closure of sagittal suture), enlarged fontanelle, blue sclerae, hypertelorism, micrognathia, arachnodactyly (fingers and toes), camptodactyly (5th fingers on both hands), joint hypermobility, hyperextensible skin, and atopic dermatitis. A head ultrasound showed hydrocephalus. Subsequent ultrasound examinations showed that the hydrocephalus had improved. There were no indications for neurosurgical intervention. The girl had left-sided conductive hearing loss. At 16 months, slight widening of the aortic root was diagnosed on ECHO. Due to dysmorphic features and congenital defects, the patient was consulted by a geneticist at age 2 years. The analysis revealed a normal female karyotype. By molecular analysis of the entire coding sequences of the *TGFβR1* and *TGFβR2* genes, the heterozygous pathogenic variant c.1582C>T (p.Arg528Cys) in the *TGFβR2* gene was detected. Genomic DNA was isolated from peripheral mononuclear blood cells. The results confirmed the clinical diagnosis of LDS, indicating type 2 specifically. The mutation c.1582C>T was not detected in the leukocyte DNAs of the unaffected parents, but (germline) mosaicism cannot be excluded. Haplotype analysis using 8 short tandem repeat markers on 6 different chromosomes confirmed paternity and thus *de novo* occurrence of c.1582C>T (19).

**Figure 3:**
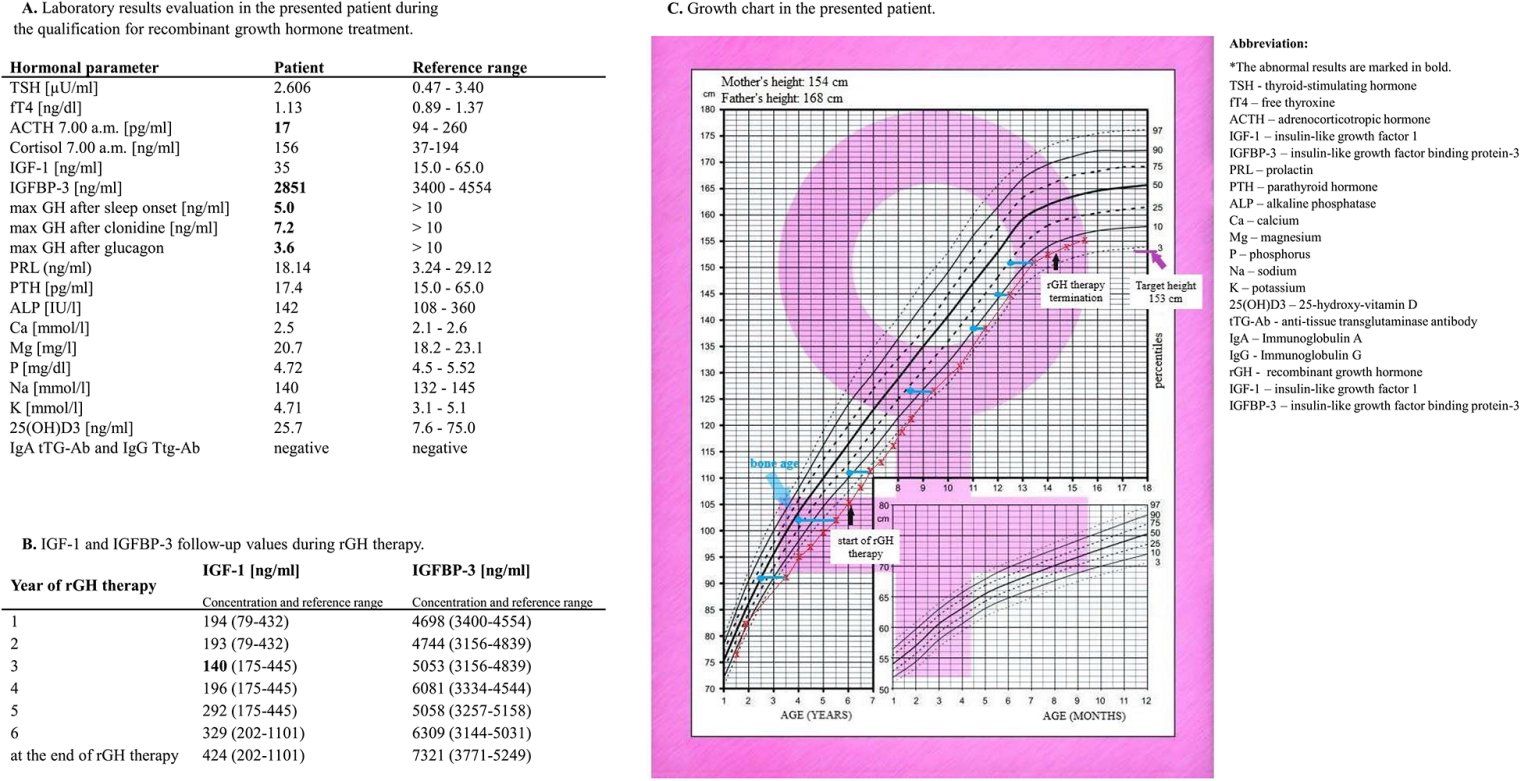
Laboratory evaluation of the patient during the qualification for rGH treatment. **(A)** IGF-1 and IGFBP-3 follow-up values during rGH therapy. **(B)** Patient growth chart. **(C)**.

At 3 years of age, a slow growth rate was observed, as her height was below the 3rd percentile. The patient was referred to an endocrinology clinic. On admission to the Department of Pediatric Endocrinology, the girl was 3.5 years old. Her height was 91 cm (htSDS −2.57), and her weight was 11 kg [<3th centile; body mass index (BMI) 13.4 kg/m^2^] (Figure 3C). Her skeletal age was 2 years and 6 months. According to the diagnostic results, the maximum GH secretion after sleep onset was 5.0 ng/ml. In stimulation tests with clonidine and glucagon, the maximum GH secretion was 7.2 and 3.6 ng/ml, respectively; therefore, partial GHD was diagnosed. On magnetic resonance imaging (MRI) of the head, we excluded a growing lesion process.

At 6 years and 2 months, the girl was still below the 3rd centile for a height of 105.5 cm, with an htSDS of −2.4 (Figure 3C). Her bone age was 4 years. Qualification for rGH therapy was initiated. Figure 3A shows the laboratory results obtained during the qualification time. When the patient was 6.5 years old, the decision to start rGH therapy was made, but intensive supervision of the cardiovascular system was needed. She was administered rGH as a subcutaneous injection. The starting dosage regimen for rGH was 0.025 mg/kg/day. The dose was adjusted during treatment according to the clinical response and laboratory data. The rGH dose was between 0.025 and 0.028 mg/kg/day, and the maximum dose was reached at 12 years. When she turned 7 years old, she reached the 3rd centile of height. She began menstruating at age 13. At 14 years and 3 months, with a satisfactory height of 154.3 cm (htSDS −1.4, equal to parental height), we terminated the rGH therapy. Her height at 16.5 years was 155 cm. During rGH therapy, the girl and her parents did not report any symptoms that might suggest side effects during the treatment. Figure 3B shows her IGF-1 and IGFBP-3 follow-up values during rGH therapy. Figure 3C shows the growth chart of the patient.

Due to dilation of the aortic root, the patient underwent regular ECHO examinations (Table 1). From the 7th year of life, therapy with 10 mg/d propranolol was started. CMR and 3D dynamic MR angiography confirmed the presence of aortic root dilatation (Figure 2). No myocardial late contrast enhancement and no contractility disorders of the left ventricle on CMR were diagnosed. Due to the diagnosis of an aneurysm in the ascending aorta, the patient was qualified for plastic surgery of the aortic root at 15 years and 8 months. The surgical outcome was satisfactory.

**Table 1:** Diameters of the aortic root (AoR) and ascending aorta (AoA) on ECHO in the patient.

	Age [years]	AoR		AoA	
		Diameter [cm]	Z-score	Diameter [cm]	Z-score
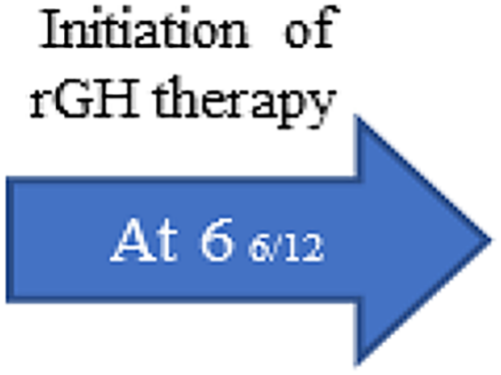	3 _2/12 _	2.3	+3.41		
	7 _5/12_	2.8	+4.01	1.7	+0.68
	8 _9/12_	2.9	+3.71	1.7	+0.03
	13	3.1	+2.78	2.1	+0.57
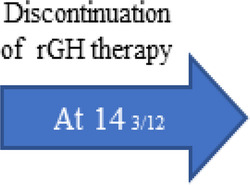	13 _10/12_	3.5	+3.85	2.1	+0.31
	14 _5/12_	4.1	+5.48	2.1	+0.18
	15 _8/12_	4.3	+5.95	2.2	+0.45
	15 _9/12_ after surgery	2.5	-0.71	2.2	+0.0
	16 _10/12_	2.3	-1.12	2.1	+0.02

The coexisting problems that influenced the patient's quality of life were several operations on lower limb deformities during LDS and severe atopic dermatitis and asthma. At 15 years of age, the girl was diagnosed with epilepsy with focal seizures. She started treatment with oxcarbazepine due to localized changes and generalized discharges recorded in the interictal electroencephalogram (EEG), accounting for her medical history (slurred speech, loss of consciousness). Through consultation with a psychologist, it was found that the patient's ability to think abstractly and logically was below the norm for her age. She had no memory disorders, but her general course of psychomotor development was inharmonious. Supplementary Figure 1 shows a photograph of the patient at age 9 months (A) and at the present time (B).

## 4 Discussion

LDS is a sporadic genetic syndrome. Current data show fewer than 4,000 patients with confirmed LDS. To our knowledge, our proband is the first patient with LDS and GHD in whom rGH therapy was introduced (11, 12). The patient in the present case was diagnosed with LDS at the early age of two years due to many disorders, such as craniofacial dysmorphism, mild aortic root dilatation, hydrocephaly, and skin and musculoskeletal disturbances (19). Because of her short stature, diagnostics were performed, and an early diagnosis of GHD was established. The laboratory results indicated partial GHD but the peripheral markers of GH action, especially IGF-1, were significantly below the normal range. The influence of coexisting comorbidities and the nutritional status of patients (BMI up to 12 years below the normal range) on IGF-1 generation should be considered. In contrast, the marked increase in IGF-1 after rGH initiation indicates that the organism has a good reaction and readiness to generate growth factors. The patient achieved satisfactory final growth after rGH therapy. More than 2 years after rGH therapy termination, the patient's height at 16.5 years was 155 cm, equal to the target height based on her parents' height. This finding suggested that the girl had recovered her growth potential at that time. A well-documented factor influencing the response to therapy is the child's age at the start of rGH treatment. Based on clinical observations, it is recommended to start rGH therapy as soon as the child has not shown a spontaneous catch-up process. This enables a better response to rGH therapy and, accordingly, a greater final height (24, 25). In addition to the growth-promoting action of rGH, the wide anabolic influence on the human body, including body composition, bone mass density, muscle mass, and strength, seems to be beneficial in patients suffering from joint hypermobility and low muscle mass. GH therapy positively influenced body composition, as shown through the patient's psychomotor development.

The right balance between the need for the substitution of GH and the safety aspects of the therapy in the presented patient must be noted. GH, physiologically, plays a key role in the development of the heart during fetal development and plays a positive role in maintaining the structure and function of the normal adult heart by stimulating cardiac growth and heart contractility. GH acts directly on myocardial growth by inducing the mRNA expression of specific proteins and facilitating cardiomyocyte proliferation (16, 26). Moreover, GH may directly act on endothelial cells through the promotion of the expression and activity of endothelial NO synthase (eNOS) (27). Patients with GHD exhibit cardiac atrophy with lower LV mass, ejection fraction, and cavity dimensions; lower cardiac output; greater peripheral vascular resistance; and lower functional capacity than healthy controls of the same age, sex, and height (28). Additionally, GHD patients exhibit accelerated development of atherosclerosis. As a result, they have a high rate of cardiovascular mortality (29). GH was found to influence collagen metabolism and change the mixture of fibrous elements in the aortic wall. GH increases muscle strength by increasing muscle mass without affecting contractile force or fiber composition (30). Therefore, GH contributes to improved myocardial performance, increasing physical activity.

Although aortic dilatation occurs in most LDS patients, the possible influence of GH therapy on aortic size must be considered. Our patient experienced a widening of the aortic root since the age of 16 months. While the aortic root increased during the administration of growth hormone, the Z-scores did not exhibit consistent increases over time. The highest increase in the diameter was observed at the age of 14 years. There are no data on the long-term effect of rGH therapy on the cardiovascular system in patients with LDS. The best experience of long-term rGH effects and safety is described in TS, where aortic dilatation may occur in early childhood (31). However, the genetic etiology of aortopathy is still under study. There are contrasting data on the influence of rGH treatment on aortic diameter in TS patients. Laroussi et al. (32) suggested that rGH therapy in TS patients had a nonsignificant influence on the aortic dimension. However, aortic dilation was observed more often in patients treated with rGH than in untreated patients (8/25 patients - 32% vs. 3/17–17.6%). A nonsignificant difference in the ascending aorta Z score was noted (32). Another study concluded that neither the history of rGH treatment nor the length of GH treatment affected the aortic diameter in patients with TS (17). Aortic root dilation in children should lead to the suspicion of connective tissue abnormalities, including LDS. Generally, patients with LDS types 1 and 2 have similar risks of aortic dissection, but males have a greater risk of aortic complications than females (33–36). Based on the follow-up of consecutive patients, the incidence of aortic root dilation ranges from 0.11–0.67 cm/year, with the most significant progression occurring in patients with LDS type 2 (36, 37). There are no clear guidelines for treating aortic aneurysms and dilatation in LDS. Surgical intervention is often recommended when the aortic diameter exceeds 4.0–4.6 cm for the aortic root and abdominal aorta, exceeds 5.0 cm for the descending thoracic aorta, or shows rapid growth (>0.5 cm/year) in any location (7, 12). Jondeau et al. (35) considered preventive replacement of the aortic root at a diameter of 45 mm or 40 mm in females with low BSA, TGFβR2 mutation, or severe extra-aortic features. Certain phenotypic factors are associated with an increased risk of aortic dissection: the presence of a TGFβR2 mutation, female sex, the presence of aortic tortuosity, hypertelorism, and translucent skin (35). The inherent weakness of the aortic wall may warrant earlier intervention, depending on the patient's family history or an evaluation of the risks and benefits of surgery (7). However, it can be assumed that a supraphysiologic increase in the IGF-1 concentration due to rGH therapy could contribute to an increase in aortic diameter. Other reports have suggested that GH does not impact aortic or ventricular size when adjusted for height or body surface area. In our own experience, strict control of plasma IGF-1 and IGFBP-3 levels ensures that they are similar to normal values for age and sex and ensures the safety of rGH therapy. The IGFBP-3 level is pivotal for the bioactivity of circulating IGF-1 (38). Blum et al. (39) found IGFBP-3 to be a more authentic discriminator of GH-dependent parameters than IGF-1 was in IGF generation tests. Careful follow-up of therapy is also necessary because concern arises with the oncogenic potential of GH. However, long-term studies in GHD children have not shown any increase in the incidence of tumors (40). Therefore, we can also suggest a significant role for the IGF-1/IGFBP-3 ratio during rGH treatment in patients with LDS syndrome (16, 41). The dose should be modified according to the IGF-1 level during treatment. In the treated patient, the IGF-1 level at the start of rGH therapy was significantly lower than normal. Her IGF-1 level was normalized during follow-up, and her IGFBP-3 level remained within or slightly above the normal range. These various biochemical results confirm the proper dosing of rGH. Indeed, additional studies are needed in the future to verify the relationship between the rGH treatment and aortic dilation progression in LDS.

## 5 Conclusions

This case report shows the results of long-term rGH therapy in a girl with LDS as a substitution therapy, which allowed us to reach a final height close to mid-parental height. The wide influence of GH on the human body, metabolic status, stabilization of the vascular wall, and muscle strength is also significant, in addition to its growth-promoting action. Because most patients with LDS experience aortic aneurysms, regular follow-up of aortic diameter is required. We do not suspect that rGH therapy had a negative impact on the dimensions of the aorta in our patient, but additional studies in the future are needed. A low IGF-1 at the GHD diagnosis and strict monitoring of the IGF-1/IGFBP-3 ratio during rGH administration were crucial in the presented patient. This is the first investigation of the effects of long-term rGH treatment on aortic dimensions in LDS.

### 5.1 Patient consent

Written informed consent was obtained from the patient's parent. Documentation was recorded in the patient's medical records.

## Data Availability

The raw data supporting the conclusions of this article will be made available by the authors, without undue reservation.

## Supplementary material

Supplementary Figure 1 Photographs of the patient at age 9 months **(A**) and at 16.5 years **(B)**.
