# Supplementary figures and images for: Case Report: Efficacy and safety of recombinant growth hormone therapy in a girl with Loeys–Dietz syndrome
Source: Front Cardiovasc Med. 2025 Jan 3;11:1377510. doi: 10.3389/fcvm.2024.1377510 (PMC11738939; doi:10.3389/fcvm.2024.1377510)

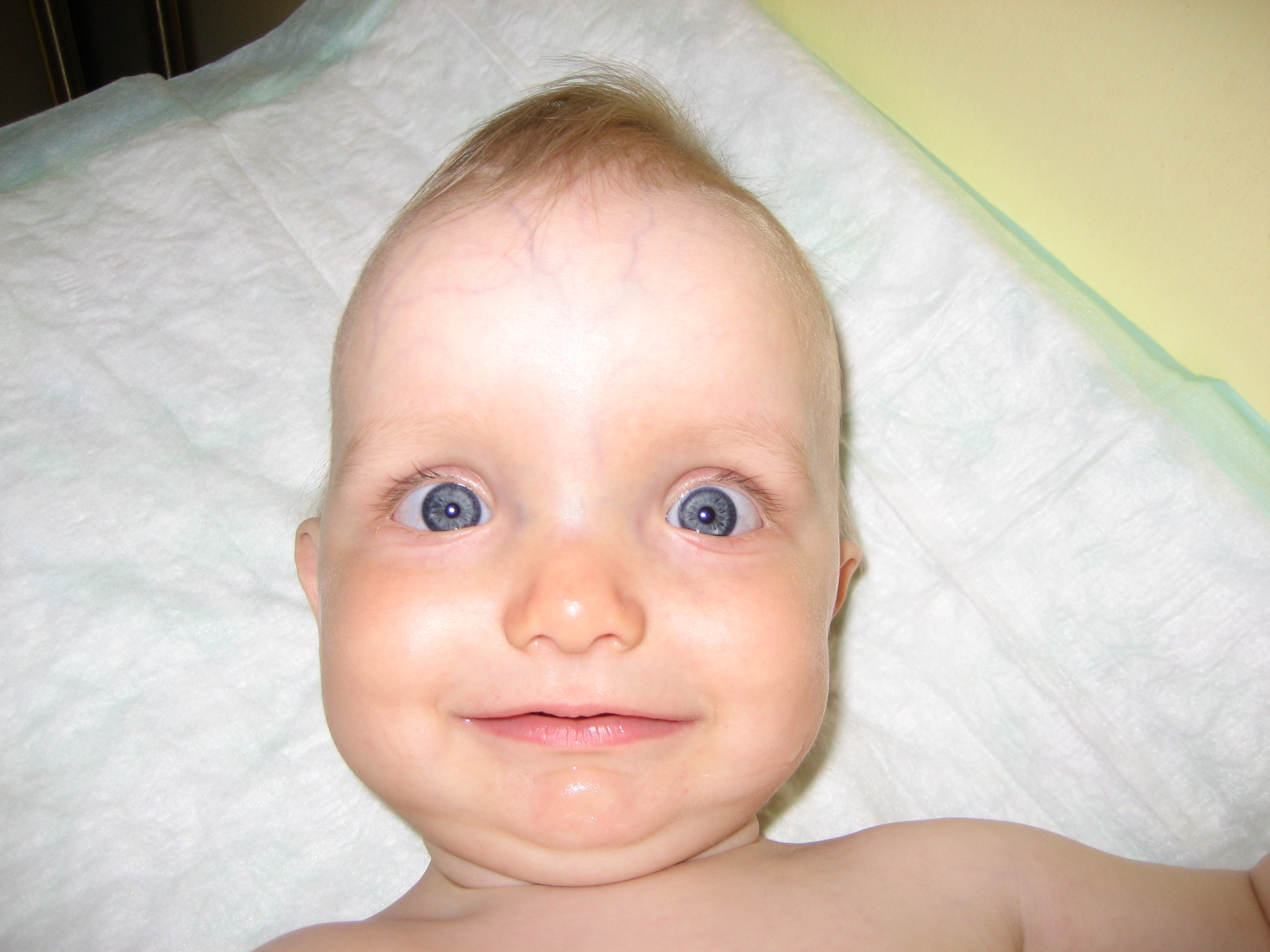

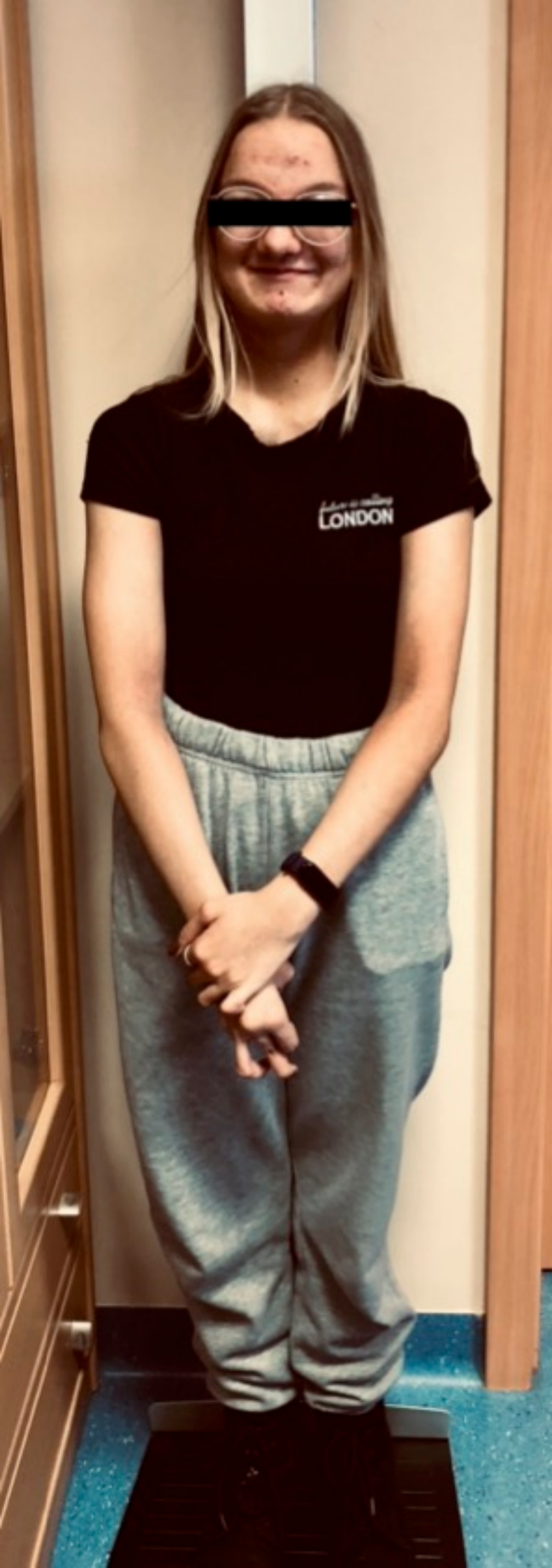

Supplement: Supplementary Figure 1 — Photographs of the patient at age 9 months (A) and at 16.5 years (B). [file Image1.pdf]
